# Factors in Time to Full Approval or Withdrawal for Anticancer Medicines Granted Accelerated Approval by the FDA

**DOI:** 10.1001/jamanetworkopen.2025.2026

**Published:** 2025-03-26

**Authors:** Ariadna Tibau, Thomas J. Hwang, Alejandra Romano, Maria Borrell, Ignasi Gich, Consolacion Molto, Aaron S. Kesselheim

**Affiliations:** 1 Program On Regulation, Therapeutics, And Law (PORTAL), Division Pharmacoepidemiology and Pharmacoeconomics, Department of Medicine, Brigham and Women’s Hospital and Harvard Medical School, Boston, Massachusetts; 2 Oncology Department, Hospital de la Santa Creu i Sant Pau, Institut d’Investigació Biomèdica Sant Pau, and Department of Medicine, Universitat Autònoma de Barcelona, Barcelona, Catalonia, Spain; 3 Cancer Innovation and Regulation Initiative, Lank Center for Genitourinary Oncology, Dana-Farber Cancer Institute, Boston, Massachusetts; 4 Division of Urological Surgery, Brigham and Women’s Hospital, Harvard Medical School, Boston, Massachusetts; 5 Vall d’Hebron Institute of Oncology (VHIO), Barcelona, Spain; 6 Medical Oncology Department, Vall d’Hebron Hospital, Barcelona, Spain; 7 Sant Pau Biomedical Research Institute (IIB Sant Pau), Barcelona, Spain; 8 CIBER Epidemiología y Salud Pública (CIBERESP), Spain; 9 Department of Clinical Epidemiology and Public Health, Hospital de la Santa Creu i Sant Pau, Barcelona, Spain; 10 R.S. McLaughlin Durham Regional Cancer Centre, Oshawa, Ontario, Canada; 11 Department of Oncology, Queen’s University, Kingston, Ontario, Canada; 12 Division of Cancer Care and Epidemiology, Queen’s Cancer Research Institute, Kingston, Ontario, Canada

## Abstract

**Importance:**

The accelerated approval pathway was developed to expedite US Food and Drug Administration (FDA) drug approval for life-threatening conditions based on changes to unvalidated surrogate measures, with conversion to regular approval then based on a required confirmatory trial. However, confirmatory trials may not be completed in a timely fashion.

**Objective:**

To analyze factors associated with time to conversion to regular approval.

**Design, setting, and participants:**

This cohort study of cancer drugs FDA-approved from 1992 to 2022 extracted pivotal and confirmatory trial characteristics, outcomes, safety data, and confirmatory study status at approval from drug labels and published reports. Clinical benefit was assessed using the European Society of Medical Oncology-Magnitude of Clinical Benefit Scale (ESMO-MCBS) for pivotal and confirmatory studies. High benefit was defined as grade A or B (for trials of curative intent) and 4 or 5 (palliative intent) and low benefit as grade C and 2 or below. Data were analyzed between August 2023 and August 2024.

**Main Outcome and Measure:**

Time to full approval or withdrawal.

**Results:**

This cohort study analyzed 102 cancer drug indications granted accelerated approval by the FDA between 1992 and 2022 and converted to regular approval by August 31, 2024. The median (IQR) time to conversion was 3.1 (1.9-4.8) years. Of these, 83 (81%) received priority review, and 27 (26%) carried boxed warnings. On the ESMO-MCBS scale, 12 of 101 pivotal trials that were scorable showed high benefit (12%), 27 intermediate benefit (27%), and 62 low benefit (61%). Twenty-one confirmatory trials (21%) were initiated after accelerated approval. Factors at the time of accelerated approval that were associated with shorter times to full approval included priority review designation, absence of boxed warnings, initiation of confirmatory studies before approval, and pivotal trials showing intermediate or high benefit on the ESMO-MCBS framework. Among the 102 confirmatory trials, 34 (33%) demonstrated significant improvements in overall survival, and 14 of 35 trials (40%) reporting on quality of life showed significant benefits. The ESMO-MCBS scale was applicable to 98 trials, with 46 (47%) scoring high clinical benefit, 29 (30%) intermediate, and 23 (23%) low. Indications with benefits in overall survival (median [IQR], 2.15 [1.40-3.38] vs 3.70 [2.33-5.78] years), quality of life (median [IQR], 2.29 [1.85-3.53] vs 4.22 [2.52-5.72] years), and high clinical benefit in confirmatory trials (median [IQR], 2.34 [1.52-3.39] vs 3.91 [2.59-6.42] years) were associated with faster conversion to full approval.

**Conclusions and Relevance:**

In this cohort study, drugs with low clinical benefit or safety concerns at accelerated approval were linked to delayed confirmatory studies, while high clinical benefit in confirmatory trials correlated with faster conversion. These associations can help guide patient decision-making around accelerated approval drugs.

## Key Points

**Question:**

What factors are associated with the fast conversion of accelerated Food and Drug Administration approval of cancer drugs to regular approval?

**Findings:**

In this cohort study of 102 cancer drug indications granted accelerated approval from 1992 to 2022 and converted to regular approval by August 2024, low clinical benefit or safety concerns at accelerated approval were associated with delayed regular approval, while high clinical benefit in confirmatory trials correlated with faster conversion.

**Meaning:**

These results suggest that the most beneficial accelerated-approval drugs tend to complete confirmatory trials soonest, indicating that delayed completion should be a cautionary signal for patients and oncologists considering these drugs.

## Teaser

This cohort study of cancer drug indications receiving accelerated approval by the US Food and Drug Administration examines factors associated with time to conversion to regular approval.

## Introduction

The US Food and Drug Administration (FDA) approves new drugs based on pivotal trials showing substantial evidence of efficacy when the benefits appear to outweigh the known risks. In response to patient advocacy for greater flexibility in the approval standard, the FDA created the accelerated approval program in 1992. This program facilitates the approval of drugs for serious or life-threatening diseases based on earlier-stage pivotal trials that demonstrate changes in unvalidated surrogate measures deemed reasonably likely by the FDA to predict clinical benefit.^1,2^ Following accelerated approval, pharmaceutical manufacturers must conduct confirmatory trials to verify clinical benefit. Failure to do so may result in removing the indication from the labeling, while successful confirmatory trials lead to conversion to regular approval.^3^

Since its inception, the program has facilitated market access to important drugs approximately 3 years earlier than traditional approval.^4^ However, delays in completing confirmatory studies have raised concerns about exposing patients to clinical and financial risks, particularly when confirmatory trials are negative, and the drug ends up being withdrawn. Policymakers have confirmed that timely completion of confirmatory trials is essential.^5^ Previous studies have shown that indications with accelerated approval that initiated confirmatory trials before approval experienced quicker transitions to traditional approval or withdrawal.^5,6,7^ In response, in December 2022, Congress approved the Consolidated Appropriations Act, granting the FDA authority to mandate confirmatory studies that are “underway prior to approval, or within a specified time period” following accelerated approval.^8^ However, other factors may affect confirmatory trial completion. To our knowledge, no comprehensive analyses have examined the factors that influence the time to full approval for cancer drug indications.

Given the importance of timely confirmatory trials for the success of the accelerated approval program, we first analyzed the factors present at the time of accelerated approval, including regulatory pathways, pivotal trial characteristics, safety concerns, clinical benefit assessments, and the initiation of confirmatory studies, which may facilitate a faster conversion to regular approval. We hypothesize that oncology drugs with higher antitumor activity and without major safety signals at the time of accelerated approval are more likely to complete confirmatory trials promptly. Second, we assessed the clinical benefit of the confirmatory studies, considering improvements in overall survival, quality of life, or clinical benefit according to scales such as the European Society of Medical Oncology–Magnitude of Clinical Benefit Scale (ESMO-MCBS), hypothesizing that oncology drugs demonstrating meaningful benefits in confirmatory trials will be more likely to complete them within the established timeframe.

## Methods

The study, based on publicly available nonidentifiable data and not involving human participants, was exempt from institutional board review and ethical approval. We adhered to the Strengthening the Reporting of Observational Studies in Epidemiology (STROBE) reporting guideline for cohort studies.

### Data Sources

In this cohort study, we explored the FDA website to identify solid and hematologic cancer drugs that received accelerated approval as either their first US approval or for a new indication from the program’s initiation in 1992 through 2022. Completion status of confirmatory trials was assessed through August 2024.^9^

### Data Extraction

For indications either converted from accelerated to regular approval or withdrawn, we documented the dates of accelerated approval, conversion,^10^ and withdrawal.^11^ Indications with ongoing accelerated approval were excluded due to the absence of a final outcome (conversion or withdrawal).

We collected information on special regulatory designations, including priority review,^12,13^ Breakthrough Therapy,^12^ and Orphan Drug Act.^14^ We gathered details regarding pivotal clinical trials, such as the number of trials supporting the proposed indication, sample sizes, trial designs (randomized vs single-group), and primary end points related to approval (overall survival vs intermediate end points, including time-to-event and non–time-to-event). For each indication, a single study was selected. When multiple pivotal trials supported the same accelerated approval indication, we prioritized those with the most robust end points, giving preference to overall survival over time-to-event end points and the latter over non–time-to-event end points. If trials had the same approval-supporting end point, we selected the trial with the most favorable results. The trial showing the most favorable results (eg, the highest response rate for single-group studies) was selected in cases with identical end points. Data on therapy type (cytotoxic, immune checkpoint inhibitors, or targeted therapies) were recorded, with genome-targeted therapies identified based on approval requiring genomic testing.^15^ Potential serious adverse reactions were extracted from original indication labels, including boxed warnings and the number of warnings and precautions listed.

We searched ClinicalTrials.gov to identify the corresponding confirmatory studies and their commencement dates. This allowed us to determine whether the trials were ongoing at the time of accelerated approval and to calculate the duration between accelerated approval and confirmatory study initiation. Data accuracy was cross-verified with an FDA-published list.^16^

### Data Synthesis and Scoring

Using FDA drug labeling^17^ and clinical trial reports, we evaluated initial and confirmatory trials using the ESMO-MCBS version 1.1 for solid tumors^18^ and version 1.0 for hematological malignant neoplasms.^19^ These tools, developed by the European Society of Medical Oncology, evaluate the effectiveness of cancer therapies based on treatment outcomes, adverse events, and quality of life to provide a comprehensive assessment of their clinical benefit. Substantial clinical benefit was defined as a grade of A or B for trials of curative intent and 4 or 5 for those of palliative intent.^18^ Intermediate benefit was categorized as grade 3 and low benefit as grade C (for curative) or 1 or 2 (for palliative).^20^ To enhance accuracy, we cross-referenced our findings with ESMO-MCBS scorecards for solid tumors^21^ and the ESMO-MCBS-H appendix for hematological malignant neoplasms,^19^ where available.

Two investigators (C.M. and M.B. or A.R. and A.T.) independently conducted sample identification, data collection, and scoring. Discrepancies were resolved through discussion.

### Statistical Analysis

Continuous variables were presented as median with interquartile ranges and compared using the Mann-Whitney U test. We first assessed whether factors at the time of accelerated approval—(1) expedited regulatory review pathways, (2) pivotal trial characteristics, (3) safety data, (4) confirmatory study status, and (5) pivotal trial clinical benefit (measured by the ESMO-MCBS)—were associated with the time to conversion to full approval. The initial analysis focused on accelerated indications, later converted to regular approval. A sensitivity analysis was subsequently conducted, including both accelerated indications with confirmed benefits and those that were withdrawn.

We also used the Mann-Whitney U test to assess whether the clinical benefit of confirmatory trials—defined by overall survival improvement, quality of life gains, or ESMO-MCBS rating—was associated with the time to full approval. A sensitivity analysis included accelerated indications with confirmed benefits and withdrawals to evaluate whether the clinical benefit of confirmatory trials was associated with time to full approval or withdrawal. Withdrawn indications were categorized as having low benefit.

For continuous variables, Spearman ρ was used to assess the correlation between the time required to demonstrate clinical benefit and these variables. Data analysis was performed in SPSS version 26.0 (IBM Corp). All tests were 2-sided, and statistical significance was defined as *P* < .05. No adjustments were made for multiple comparisons.

## Results

From 1992 to 2022, the FDA granted accelerated approval to 166 indications for 113 anticancer solid and hematologic tumor drugs. As of August 31, 2024, excluding 34 ongoing accelerated approvals (20%), our study cohorts comprise 102 indications (61%) that were converted to full approval and 30 (18%) that were withdrawn. eTables 1 and 2 in Supplement 1 provide detailed information on product indications, as well as the dates of accelerated approval, full approval, and withdrawals. The median (IQR) time between accelerated approval and conversion to regular approval was 3.10 (1.90-4.83) years, and between accelerated approval and withdrawal was 3.82 (2.78-7.89) years.

Table 1 provides a summary of the key characteristics of the indications and pivotal trials for the 102 accelerated approvals that were converted to regular approval. These approvals were associated with priority review in 83 cases (81%), Breakthrough Therapy designation in 37 out of 59 cases (63%), and Orphan Drug Act designation in 65 cases (64%). Sixty-one (60%) were for initial indications. Sixty-seven (66%) of the accelerated approved indications were for solid tumor drugs and 35 (34%) for hematologic cancers. Thirty-seven approvals (36%) were for genome-targeted therapies, and 23 (23%) were for immune checkpoint inhibitors. For safety, 27 indications (26%) included a boxed warning at the time of accelerated approval (median [IQR] warnings, 2 [1-3]). The median number of warnings and precautions in the labeling was 6 (4-8).

**Table 1.** Factors at Accelerated Approval Associated With Time to Full Approval for Anticancer Drugs, 1992-2022

Cancer drug indications and pivotal trials	Trials, No. (%)	Differences in approval times, median (IQR), y	*P* value^a^
No. of patients, median (IQR) (n = 102)	143 (87.75-235.23)	NA	.13^b^
Response rate, median (IQR), % (n = 77)	38 (24-55)	NA	.26^c^
Warnings and precautions, median (IQR), No. (n = 102)	6 (4-8)	NA	.11^d^
Priority review (n = 102)			
Yes	83 (81)	2.90 (1.86-4.34)	.002
No	19 (19)	5.11 (3.21-8.07)	
Breakthrough Therapy designation (n = 59)^e^			
Yes	37 (63)	2.33 (1.43-3.45)	.94
No	22 (37)	2.63 (1.68-3.72)	
Orphan Drug Act designation (n = 102)			
Yes	65 (64)	3.40 (2.22-5.24)	.054
No	37 (36)	2.61 (1.68-4.31)	
Tumor type (n = 102)			
Solid Cancer	67 (66)	2.93 (1.86-4.54)	.11
Hematologic Cancer	35 (34)	3.59 (2.20-6.37)	
Type of indication (n = 102)			
Initial	61 (60)	3.81 (2.06-5.22)	.13
Supplemental	41 (40)	2.66 (1.89-4.15)	
Companion diagnostic (n = 102)			
Yes	36 (35)	2.81 (1.97-3.73)	.16
No	66 (65)	3.55 (1.89-5.31)	
Genome-targeted drugs (n = 102)			
Yes	37 (36)	2.93 (2.20-4.16)	.45
No	65 (64)	3.43 (1.85-5.38)	
Immunotherapy (n = 102)			
Yes	23 (23)	3.14 (1.84-5.54)	.49
No	79 (77)	3.07 (2.11-5.11)	
No. of trials supporting approval (n = 102)			
1	78 (76)	3.13 (2.08-4.65)	.60
>1	24 (24)	2.98 (1.90-7.25)	
Study design (n = 102)			
SAT	70 (69)	2.98 (2.14-4.78)	.80
RCT	32 (31)	3.53 (1.85-5.05)	
Masking (n = 32)			
Open-label	24 (75)	3.52 (1.84-5.05)	.69
Double-masked	8 (25)	3.62 (3.05-5.21)	
Time to event end point leading (n = 102)			
Yes	16 (16)	3.07 (1.31-5.43)	.58
No	86 (84)	3.11 (2.06-4.78)	
Confirmatory trial ongoing at time of accelerated approval (n = 102)			
Yes	81 (79)	2.78 (1.84-4.16)	<.001
No	21 (21)	5.59 (3.62-8.23)	
Boxed warnings (n = 102)			
Yes	27 (26)	4.61(2.60-8.07)	<.001
No	75 (74)	2.90 (1.86-4.29)	
Low clinical benefit ESMO-MCBS (n = 101)^f^			
Yes	62 (61)	3.81 (2.11-5.85)	.03
No	39 (39)	2.37 (1.90-3.99)	

Abbreviation: ESMO-MCBS, European Society for Medical Oncology Magnitude of Clinical Benefit; NA, not applicable.

^a^ The Mann-Whitney U test assessed whether application and trial characteristics in the accelerated approval pathway were associated with time from accelerated approval to full approval.

^b^ Spearman ρ = −0.15 for correlations between continuous variables and time to demonstrate clinical benefit.

^c^ Spearman ρ = −0.11 for correlations between continuous variables and time to demonstrate clinical benefit.

^d^ Spearman ρ = 0.16 for correlations between continuous variables and time to demonstrate clinical benefit.

^e^ Breakthrough Therapy Designation came into effect in July 2012.

^f^ ESMO-MCBS was applicable to 101 of the 102 pivotal trials. Among these, 12 trials (12%) demonstrated a high clinical benefit, 27 trials (27%) intermediate, and 62 (61%) low clinical benefit.

The 102 accelerated approval indications later converted to regular approval were associated with 102 pivotal trials, of which 86 (84%) used a surrogate measure of response rate as the primary end point, while 10 (10%) used progression-free survival, 5 (5%) used disease-free survival, and 1 (1%) used overall survival. The median (IQR) sample size was 143.00 (87.75-235.23). When accelerated approval was granted, 21 confirmatory trials (21%) had not started.

The ESMO-MCBS was applicable to 101 out of 102 (99%) pivotal trials supporting accelerated approval. Among these, 12 (12%) were rated as having high benefit, 27 (27%) intermediate clinical benefit, and 62 (61%) as low benefit. Comparable percentages were observed when withdrawal indications were included in the regular cohort (eTable 3 in Supplement 1).

### Clinical Benefit of Confirmatory Trials

Among the 102 confirmatory trials, 34 (33%) demonstrated significant changes to overall survival. Additionally, of the 35 trials that reported results on quality of life, 14 (40%) also demonstrated significant results.

In terms of confirmatory trials, ESMO-MCBS was applicable to 98 of the 102 trials (96%). Among these, 46 trials (47%) demonstrated a high clinical benefit, 29 trials (30%) intermediate, and 23 trials (23%) low (Table 2). Comparable percentages were observed when withdrawal indications were included in the regular cohort (eTable 4 in Supplement 1).

**Table 2.** Association of Clinical Benefit of Confirmatory Trials With Time to Full Approval for Anticancer Drugs Granted Accelerated Approval, 1992-2022

Confirmatory trials	FDA approval, No. (%)	Differences in approval times, median (IQR), y	*P* value^a^
Overall survival benefit^b^ (n = 102)			
Yes	34 (33)	2.15 (1.40-3.38)	<.001
No	68 (67)	3.70 (2.33-5.78)	
Quality of life benefit^c^ (n = 35)			
Yes	14 (40)	2.29 (1.85-3.53)	.01
No	21(60)	4.22 (2.52-5.72)	
ESMO-MCBS high-clinical benefit^d^ (n = 98)			
Yes	46 (47)	2.34 (1.53-3.39)	<.001
No	52 (53)	3.91 (2.59-6.42)	

Abbreviations: ESMO-MCBS, European Society for Medical Oncology Magnitude of Clinical Benefit; FDA, US Food and Drug Administration.

^a^ The Mann-Whitney U test evaluated whether overall survival, quality of life, and ESMO-MCBS clinical benefit were associated with time from accelerated to full approval.

^b^ Overall survival benefit as documented in FDA-approved labeling.

^c^ Quality of life data were extracted from articles reporting confirmatory trial results. At the time of regular approval conversion, only 35 pivotal trials included quality-of-life data.

^d^ ESMO-MCBS was applicable to 98 of the 102 trials (96%). Among these, 46 trials (47%) demonstrated a high clinical benefit; 29 trials (30%) intermediate, and 23 trials (23%) low clinical benefit.

### Association Between Time to Conversion and Characteristics at Accelerated Approval

**Figure 1.**
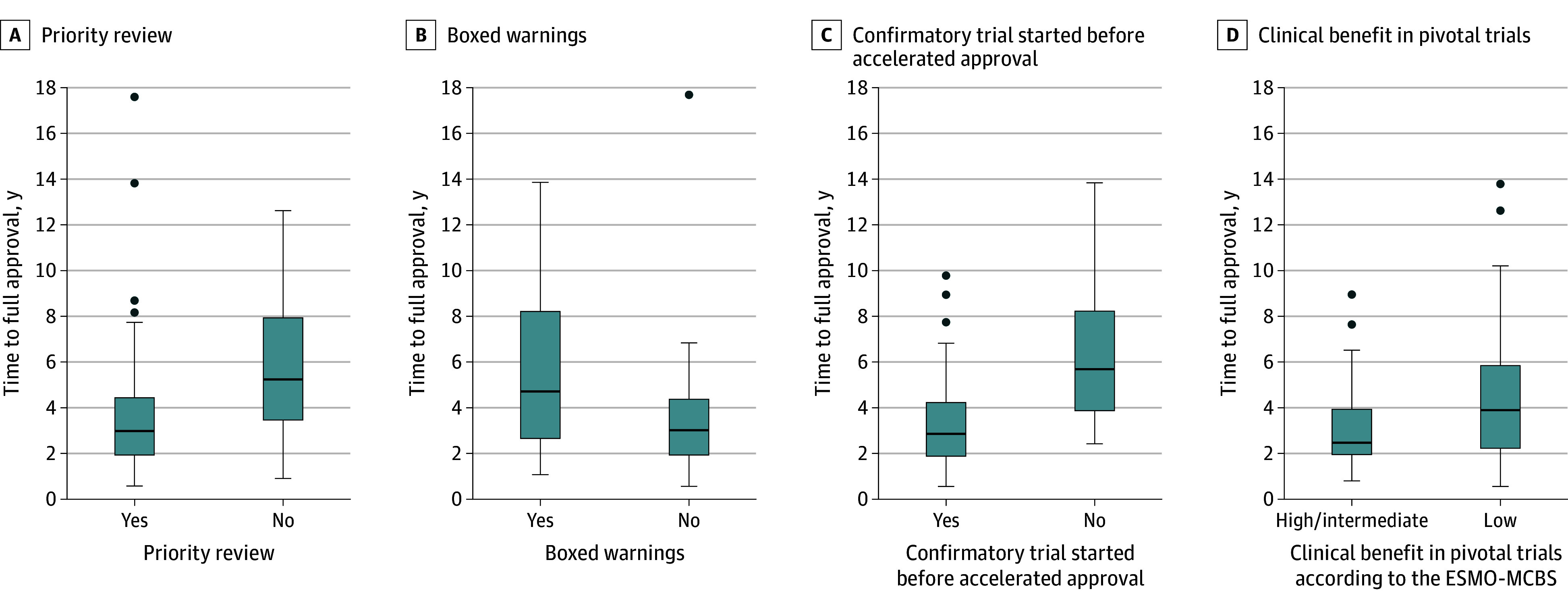
Factors at Accelerated Approval Associated With Time to Full Approval for Anticancer Medicines Granted Accelerated Approval, 1992-2022 The black line represents the median, the colored bars indicate the quartiles, the whiskers show the extremes, and outliers are marked with dots.

Table 1 and Figure 1 show associations at the time of accelerated approval with the time to achieve regular approval. eTable 3 in Supplement 1 provides similar associations, also including outcomes of either conversion to regular approval or withdrawal.

Accelerated approval indications that received priority review reached completion faster than those under regular review (median [IQR] difference in approval time, 2.90 [1.86-4.34] vs 5.11 [3.21-8.07] years; *P* = .002) (Table 1). This difference remained significant even when withdrawn indications were included (3.03 [2.11-4.54] vs 6.33 [3.12-8.23] years; *P* = .002) (eTable 3 in Supplement 1).

Indications with ongoing confirmatory trials at the time of accelerated approval were also associated with shorter completion times compared with those without a confirmatory trial ongoing at the time of accelerated approval. This difference was significant for indications that converted to regular approval (2.78 [1.84-4.16] vs 5.59 [3.62-8.23] years; *P* < .001) and persisted when withdrawn indications were included (2.98 [1.88-4.36] vs 5.98 [3.25-8.54] years; *P* < .001).

Indications with 1 or more boxed warnings at accelerated approval took longer to achieve full approval (4.61 [2.60-8.07] vs 2.90 [1.86-4.29] years; *P* < .001). Significance persisted after including withdrawn indications (4.61 [2.72-8.23] vs 3.03 [1.87-4.51] years; *P* < .001).

At the time of accelerated approval, pivotal trials demonstrating low benefit according to the ESMO-MCBS exhibited longer completion times (3.81 [2.11-5.85] vs 2.37 [1.90-3.99] years; *P* = .03). This significance persisted even after incorporating withdrawn indications (3.81 [2.50-6.37] vs 2.58 [1.90-3.99] years; *P* = .009).

### Association Between Time to Conversion and Clinical Benefit of Confirmatory Trials

**Figure 2.**
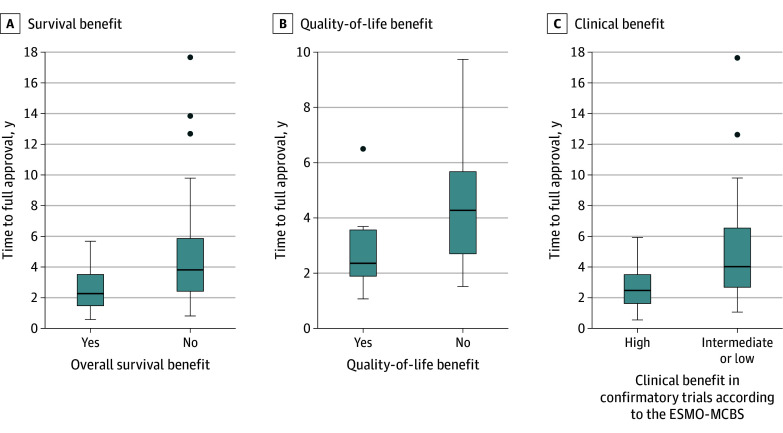
Association of Clinical Benefit of Confirmatory Trials Associated With Time to Full Approval for Anticancer Medicines Granted Accelerated Approval, 1992-2022 The black line represents the median, the colored bars indicate the quartiles, the whiskers show the extremes, and outliers are marked with dots.

Table 2 and Figure 2 display associations between the clinical benefit of confirmatory trials and the time to achieve regular approval. eTable 4 in Supplement 1 provides similar associations, also including outcomes of either conversion to regular approval or withdrawal.

Shorter times to confirmatory trial completion were associated with confirmatory trials showing high clinical benefit (median [IQR], 2.34 [1.53-3.39] vs 3.91 [2.59-6.42] years; *P* < .001) (Table 2). Similarly, shorter times to regular approval were associated with indications with overall survival benefit (median [IQR], 2.15 [1.40-3.38] vs 3.70 [2.33-5.78] years; *P* < .001) or quality-of-life benefit in confirmatory trials (median [IQR], 2.29 [1.85-3.53] vs 4.22 [2.52-5.72] years; *P* = .01). When withdrawn indications were included, the association remained between the timing of accelerated approval conversion to regular approval and high clinical benefit ratings according to ESMO-MCBS (median [IQR], 2.34 [1.52-3.39] vs 3.85 [2.62-6.53] years; *P* = .001). This significance also held for overall survival (median [IQR], 2.15 [1.40-3.38] vs 3.81 [2.47-6.44] years; *P* < .001) and quality of life (median [IQR], 2.34 [1.86-3.59] vs 4.22 [2.66-5.59] years; *P* = .03).

## Discussion

Ensuring timely initiation and completion of postapproval trials is a crucial aspect of the FDA’s accelerated approval program. Our analysis identified several additional factors associated with faster completion of confirmatory trials for cancer drugs granted accelerated approval: priority review designations, the absence of serious safety concerns indicated by boxed warnings at the time of accelerated approval, the initiation of confirmatory studies prior to accelerated approval, and approval based on pivotal trials with a benefit threshold of grade 3 or higher according to the ESMO-MCBS. Patients receiving accelerated approval drugs with these characteristics known at the time of accelerated approval were more likely to be treated with medications subsequently shown to be effective for their condition and to achieve conversion to regular approval more promptly.

We also found that accelerated approval of cancer drugs with more meaningful benefits in confirmatory trials, such as an overall survival benefit and quality-of-life benefit, as well as those with substantial clinical benefit according to ESMO-MCBS, underwent faster completion of their confirmatory studies. These results are reassuring, confirming substantial time savings in terms of earlier availability of beneficial drugs to patients. However, a quarter of the drugs granted accelerated approval showed low clinical benefit according to the ESMO-MCBS framework at the time of conversion to full approval and remained on the market for extended periods—often over 3 years—without verification.

Previous research on the time to full approval of new oncology treatments has primarily focused on the link between the initiation of confirmatory trials and the time to traditional approval or withdrawal.^5,6,7^ Consistent with prior findings, our study shows that trials already under way at initial approval tend to be completed more quickly. Our results also align with recent legislative efforts, such as the Consolidated Appropriations Act, which grants the FDA greater authority to enforce timely completion of confirmatory trials in the accelerated approval pathway.^8^ Our current analysis expands on this by identifying additional factors influencing trial completion. Drugs qualifying for priority review—a designation for treatments aimed at improving outcomes for serious conditions, with a 6-month review timeline instead of the standard 10 months—tend to complete confirmatory trials faster. By contrast, drugs with early safety concerns, such as boxed warnings, are more likely to remain on the market for extended periods without verified clinical benefits, exposing patients to treatments with uncertain benefit-risk profiles.

Recent FDA guidance recommends that trials supporting accelerated approval demonstrate the “clinical meaningfulness” of treatment effects beyond statistical significance.^22^ However, specific definitions for these criteria remain undefined. Our findings suggest that early indicators of clinical benefit, such as the ESMO-MCBS tool, could refine the approval pathway by prioritizing drugs with substantial advantages over existing treatments. This approach may enable more timely regulatory decisions for drugs initially approved on limited evidence and support patients and physicians in prioritizing high-value cancer treatments.

### Policy Implications

Our study identifies certain areas for improving the accelerated approval program. First, while improvements in surrogate measures currently support nearly all accelerated approval and account for more than half of conversions to full approval,^23^ growing evidence suggests that many end points, such as progression-free and disease-free survival, do not reliably correlate with overall survival in most clinical contexts.^24^ This raises questions about whether the pathway should continue to support treatments based on end points that lack validated surrogacy.^25^ Second, the FDA should consider whether this pathway should be restricted to drugs with a meaningful level of activity, no initial signs of serious adverse effects, or meaningful clinical benefit as defined by the ESMO-MCBS, with at least intermediate benefit at the time of accelerated approval. Third, the agency should not hesitate to use its expanded statutory authority and require that confirmatory trials be under way, or even with recruitment completed, at the time of accelerated approval with annual public reporting of confirmatory trial status, as mandated by recent legislation.^8^ Fourth, confirmatory trials should verify the clinical benefit in terms of benefits in overall survival, quality of life, or demonstrating a substantial clinical benefit according to validated frameworks of clinical benefit.^26^ Aligning payer reimbursement with FDA confirmatory study deadlines may help ensure the timely completion of confirmatory studies.^27^ Finally, the cost of accelerated approval drugs must be consistent with their clinical benefit,^28^ especially when that benefit remains uncertain.

### Limitations

This study had several limitations. First, some recent accelerated approvals, granted only a year or 2 before the end of the study period, did not contribute to the data. Second, we excluded drugs with ongoing confirmatory trials, which limits the generalizability of the study. Third, we expanded the definition of clinical benefit beyond the original (version 1.0)^29^ and current (version 1.1)^18^ versions of the ESMO-MCBS scale by introducing a third category for drugs with intermediate or moderate benefit, as previously described.^20^ This adjustment was necessary because only 12% of pivotal trials supporting accelerated approvals demonstrated high clinical benefit, limiting the scope of our statistical analysis. ESMO-MCBS scores of 3 or higher are associated with positive health technology assessments, highlighting their role in identifying therapies that offer moderate benefits and warrant reimbursement.^20^ Finally, due to limitations in publicly available data, we encountered challenges in identifying certain confirmatory studies.

## Conclusions

Drugs with shorter intervals from accelerated approval to completion of confirmatory studies demonstrated higher pre–accelerated approval clinical benefits and substantial clinical benefits in confirmatory trials. In these cases, important drugs are being made available earlier for the benefit of patients. However, for the remaining indications, the FDA must work to minimize the duration during which patients and physicians use drugs approved via accelerated pathway without robust data on their efficacy and safety. Efforts to expedite drug development and approval, especially for life-threatening diseases like cancer must continue. However, these efforts should be followed by rigorous confirmatory data to refine the indications and prioritize therapies with clinically meaningful outcomes.

## Notes

Supplement 1. **eTable 1.** Accelerated Approvals for Malignant Hematology and Oncology Products That Verified Benefit **eTable 2.** Accelerated Approvals for Malignant Hematology and Oncology Products That Were Withdrawn **eTable 3.** Factors at Accelerated Approval Associated With Time to Full Approval and Withdrawal for Anticancer Drugs, 1992-2022 **eTable 4.** Clinical Benefit of Confirmatory Trials Associated With Time to Full Approval and Withdrawal for Anticancer Drugs Granted Accelerated Approval, 1992-2022

Supplement 2.
Data Sharing Statement
