# Supplementary material for: Factors in Time to Full Approval or Withdrawal for Anticancer Medicines Granted Accelerated Approval by the FDA
Source: JAMA Netw Open. 2025 Mar 26;8(3):e252026. doi: 10.1001/jamanetworkopen.2025.2026 (PMC11947834; doi:10.1001/jamanetworkopen.2025.2026)
Supplement: Supplement 1. — eTable 1. Accelerated Approvals for Malignant Hematology and Oncology Products That Verified Benefit eTable 2. Accelerated Approvals for Malignant Hematology and Oncology Products That Were Withdrawn eTable 3. Factors at Accelerated Approval Associated With Time to Full Approval and Withdrawal for Anticancer Drugs, 1992-2022 eTable 4. Clinical Benefit of Confirmatory Trials Associated With Time to Full Approval and Withdrawal for Anticancer Drugs Granted Accelerated Approval, 1992-2022 [file jamanetwopen-e252026-s001.pdf]

Supplemental Online Content

Tibau A, Hwang TJ, Romano A, et al. Factors in time to full approval or withdrawal for anticancer medicines granted accelerated approval by the FDA. *JAMA Netw Open.* 2025;8(3):e252026. doi:10.1001/jamanetworkopen.2025.2026

eTable 1. Accelerated Approvals for Malignant Hematology and Oncology Products That Verified Benefit

eTable 2. Accelerated Approvals for Malignant Hematology and Oncology Products That Were Withdrawn

eTable 3. Factors at Accelerated Approval Associated With Time to Full Approval and Withdrawal for Anticancer Drugs, 1992-2022

eTable 4. Clinical Benefit of Confirmatory Trials Associated With Time to Full Approval and Withdrawal for Anticancer Drugs Granted Accelerated Approval, 1992–2022

This supplemental material has been provided by the authors to give readers additional information about their work

© 2025 Tibau A et al. *JAMA Network Open*.

**Supplementary Table 1.** Accelerated Approvals for Malignant Hematology and Oncology Products That Verified Benefit			
**Product**	**Original Accelerated Approval Indication**	**Date of AA**	**Date of RA**
Alemtuzumab (Campath)	Refractory B-cell CLL	5/7/2001	9/19/2007
Alectinib (Alecensa)	Refractory ALK+ metastatic NSCLC	12/11/2015	11/6/2017
Amivantamab-vmjw (Rybrevant)	Refractory advanced NSCLC with EGFR exon 20 insertion mutations	5/21/2021	3/1/2024
Anastrozole (Arimidex)	Adjuvant, postmenopausal, HR+ early BC	9/5/2002	9/16/2005
Asciminib (Scemblix)	Refractory Ph+ CML in CP	10/29/2021	10/12/2022
Avelumab (Bavencio)	Refractory advanced UC	5/9/2017	6/30/2020
Avelumab (Bavencio)	Metastatic MCC	3/23/2017	9/6/2023
Bevacizumab (Avastin)	Refractory glioblastoma	5/5/2009	12/5/2017
Bicalutamide (Casodex)	Advanced prostate cancer	10/4/1995	12/12/1997
Blinatumomab (Blincyto)	Refractory Ph- or B-cell precursor ALL	12/3/2014	7/11/2017
Blinatumomab (Blincyto)	CD19+ B-cell precursor ALL in first/second complete remission with MRD ≥0.1%	3/29/2018	6/20/2023
Bortezomib (Velcade)	Refractory MM	5/13/2003	3/25/2005
Bosutinib (Bosulif)	Newly diagnosed Ph+ CML in CP	12/19/2017	5/14/2021
Brentuximab vedotin (Adcetris)	Refractory HL	8/19/2011	8/17/2015
Brentuximab vedotin (Adcetris)	Refractory systemic anaplastic large cell lymphoma	8/19/2011	3/20/2018
Brigatinib (Alunbrig)	Refractory ALK+ metastatic NSCLC	4/28/2017	5/22/2020
Capecitabine (Xeloda)	Refractory Metastatic BC	4/30/1998	9/7/2001
Capmatinib (Tabrecta)	Metastatic NSCLC with MET exon 14 skipping alterations	5/6/2020	8/10/2022
Carfilzomib (Kyprolis)	Refractory MM	7/20/2012	1/21/2016
Cemiplimab-rwlc (Libtayo)	Refractory metastatic BCC	2/9/2021	4/28/2023
Ceritinib (Zykadia)	Refractory ALK+ metastatic NSCLC	4/29/2014	5/26/2017
Cetuximab (Erbitux)	As a single agent, EGFR+ refractory metastatic CRC	2/12/2004	10/2/2007
Cetuximab (Erbitux)	EGFR+ refractory metastatic CRC	2/12/2004	7/6/2012
Clofarabine (Clolar)	Pediatric, refractory ALL	12/28/2004	7/18/2022
Crizotinib (Xalkori)	Refractory ALK+ metastatic NSCLC	8/26/2011	11/20/2013
Dabrafenib (Tafinlar)	Unresectable or metastatic melanoma with BRAF V600E/K mutations	1/9/2014	11/20/2015
Daratumumab (Darzalex)	Refractory MM	11/16/2015	11/21/2016
Dasatinib (Sprycel)	Refractory CML	6/28/2006	5/21/2009
Dasatinib (Sprycel)	Newly diagnosed Ph+ CML in CP	10/28/2010	8/12/2015

© 2025 Tibau A et al. *JAMA Network Open*.

Denileukin diftitox (Ontak)	CD25+ refractory CTCL	2/5/1999	10/15/2008
Docetaxel (Taxotere)	Refractory locally advanced or metastatic BC	5/14/1996	6/22/1998
Dostarlimab-gxly (Jemperli)	Refractory dMMR advanced EC	4/22/2021	2/9/2023
Enfortumab vedotin-ejfv (Padcev)	Refractory advanced UC	12/18/2019	7/9/2021
Erdafitinib (Balversa)	Refractory advanced UC, with FGFR3/FGFR2 genetic alterations	4/12/2019	1/19/2024
Everolimus (Afinitor)	TSC who have SEGA not candidates for curative surgical resection	10/29/2010	1/29/2016
Everolimus (Afinitor)	Renal angiomyolipoma associated with TSC	4/26/2012	2/18/2016
Fam-trastuzumab deruxtecan-nxki (Enhertu)	Refractory metastatic HER2+	12/20/2019	5/4/2022
Ibritumomab tiuxetan (Zevalin)	Refractory low-grade follicular or transformed B-cell NHL	2/19/2002	9/3/2009
Ibrutinib (Imbruvica)	Refractory CLL	2/12/2014	7/28/2014
Imatinib mesylate (Gleevec)	CML in blast crisis, AP, or in CP after failure of interferon-alpha	5/10/2001	12/8/2003
Imatinib mesylate (Gleevec)	KIT (CD117) positive unresectable and/or metastatic GIST	2/1/2002	9/26/2008
Imatinib mesylate (Gleevec)	Newly diagnosed adults with Ph+ CML	12/20/2002	5/27/2009
Imatinib mesylate (Gleevec)	Pediatric patients, refractory PH+ CP CML	5/20/2003	9/27/2006
Imatinib mesylate (Gleevec)	Newly diagnosed Ph+ CML in pediatric patients	9/27/2006	4/1/2011
Imatinib mesylate (Gleevec)	Adjuvant KIT+ GIST	12/19/2008	1/31/2012
Irinotecan hcl trihydrote (Camptosar)	Refractory metastatic CRC	6/14/1996	10/22/1998
Lapatinib (Tykerb)	Post-menopausal HR+/HER2+ metastatic BC	1/29/2010	12/6/2018
Letrozole (Femara)	Adjuvant, postmenopausal women, HR+ BC, extended therapy.	10/29/2004	4/30/2010
Letrozole (Femara)	Adjuvant, postmenopausal women, HR+ BC	12/28/2005	4/30/2010
Lenvatinib (Lenvima)	Refractory advanced endometrial carcinoma not MSI-H or dMMR	9/17/2019	7/21/2021
Liposomal cytarabine (Depocyt)	Lymphomatous meningitis	4/1/1999	4/19/2007
Liposomal doxorubicin (Doxil)	Refractory AIDS-related Kaposi's sarcoma	11/17/1995	6/10/2008
Liposomal doxorubicin (Doxil)	Refractory metastatic ovarian carcinoma	6/28/1999	1/28/2005
Lorlatinib (Lorbrena)	Refractory ALK+ metastatic NSCLC	11/2/2018	3/3/2021
Mirvetuximab soravtansine-gynx (Elahere)	Refractory FRα+, ovarian, fallopian tube, or primary peritoneal cancer	11/14/2022	3/22/2024
Nelarabine (Arranon)	Refractory T-cell ALL or T-cell lymphoblastic lymphoma	10/28/2005	7/31/2019
Nilotinib (Tasigna)	Newly diagnosed, Ph+ CML in CP	6/17/2010	1/27/2015
Nilotinib (Tasigna)	Refractory CP and AP Ph+ CML	10/29/2007	1/14/2011
Nivolumab (Opdivo)	Refractory unresectable or metastatic melanoma	12/22/2014	3/7/2019
Nivolumab (Opdivo)	BRAF V600 wild-type unresectable or metastatic melanoma	9/30/2015	3/7/2019
Nivolumab (Opdivo)	Refractory advanced UC	2/2/2017	8/19/2021

© 2025 Tibau A et al. *JAMA Network Open*.

Ofatumumab (Arzerra)	Refractory CLL	10/26/2009	4/17/2014
Olaparib (Lynparza)	Refractory germline BRCA-mutated advanced ovarian cancer	12/19/2014	8/17/2017
Omacetaxine mepesuccinate (Synribo)	Refractory CML with CP or AP	10/26/2012	2/10/2014
Osimertinib (Tagrisso)	Refractory metastatic EGFR T790M mutation-positive NSCLC.	11/13/2015	3/30/2017
Oxaliplatin (Eloxatin)	Refractory metastatic CRC	8/9/2002	1/9/2004
Palbociclib (Ibrance)	Postmenopausal HR+ newly diagnosed metastatic BC	2/3/2015	3/31/2017
Panitumumab (Vectibix)	Refractory EGFR+ metastatic CRC	9/27/2006	5/23/2014
Pembrolizumab (Keytruda)	Refractory unresectable or metastatic melanoma	9/4/2014	12/18/2015
Pembrolizumab (Keytruda)	Refractory metastatic PD-L1 + NSCLC	10/2/2015	10/24/2016
Pembrolizumab (Keytruda)	Refractory HNSCC	8/5/2016	6/10/2019
Pembrolizumab (Keytruda)	Refractory classical HL	3/14/2017	10/14/2020
Pembrolizumab (Keytruda)	Newly diagnosed metastatic non-squamous NSCLC	5/10/2017	8/20/2018
Pembrolizumab (Keytruda)	Advanced UC ineligible for cisplatin-containing chemotherapy	5/18/2017	8/31/2021
Pembrolizumab (Keytruda)	Refractory metastatic, MSI-H or dMMR solid tumors or CRC	5/23/2017	3/28/2023
Pembrolizumab (Keytruda)	Refractory primary mediastinal large B-cell lymphoma	6/13/2018	10/14/2020
Pembrolizumab (Keytruda)	Refractory PD-L1+ metastatic cervical cancer	6/12/2018	10/13/2021
Pembrolizumab (Keytruda)	Recurrent locally advanced or metastatic MCC	12/19/2018	10/12/2023
Pembrolizumab (Keytruda)	Refractory advanced endometrial carcinoma not MSI-H or dMMR	9/17/2019	7/21/2021
Pembrolizumab (Keytruda)	Locally recurrent unresectable or metastatic PD-L1+ TNBC	11/13/2020	7/26/2021
Pembrolizumab (Keytruda)	Refractory HCC	11/9/2018	1/25/2024
Pemetrexed disodium (Alimta)	Newly diagnosed metastatic non-squamous NSCLC	6/4/2018	1/30/2019
Pemetrexed disodium (Alimta)	Refractory metastatic NSCLC	8/19/2004	7/2/2009
Pemetrexed disodium (Alimta)	Newly diagnosed or refractory non-squamous NSCLC	9/26/2008	7/2/2009
Pertuzumab (Perjeta)	Neoadjuvant HER2+ BC treatment	9/30/2013	12/20/2017
Pomalidomide (Pomalyst)	Refractory MM	2/8/2013	4/23/2015
Polatuzumab vedotin- piiq (Polivy)	Refractory DLBCL	6/10/2019	4/19/2023
Ponatinib (Iclusig)	Refractory CML in CP, AP, or BP or Refractory Ph+ ALL	12/14/2012	11/28/2016
Pralsetinib (Gavreto)	Metastatic RET fusion+ NSCLC	9/4/2020	8/9/2023
Rucaparib (Rubraca)	BRCA-mutated refractory advanced ovarian cancer	12/19/2016	4/6/2018
Sacituzumab govitecan-hziy (Trodelvy)	Refractory metastatic TNBC	4/22/2020	4/7/2021
Selinexor (Xpovio)	Refractory MM	7/3/2019	12/18/2020
Selpercatinib (Retevmo)	Metastatic RET fusion+ NSCLC	5/8/2020	9/21/2022

© 2025 Tibau A et al. *JAMA Network Open*.

Selpercatinib (Retevmo)	Advanced RET fusion+ thyroid cancer	5/8/2020	6/12/2024
Sunitinib maleate (Sutent)	Advanced RCC	1/26/2006	2/2/2007
Thalidomide (Thalomid)	Newly diagnosed MM	5/25/2006	6/19/2014
Temozolomide Temodar	Adults with refractory anaplastic astrocytoma	8/11/1999	3/15/2005
Tepotinib (Tepmetko)	Metastatic NSCLC with MET exon 14 skipping alterations	2/3/2021	2/15/2024
Tisotumab vedotin-tftv (Tivdak)	Refractory metastatic cervical cancer	9/20/2021	4/29/2024
Trametinib (Mekinist)	Unresectable or metastatic melanoma with BRAF V600E/K mutations	1/8/2014	11/20/2015
Venetoclax (Venclexta)	Refractory CLL with 17P deletion	4/11/2016	6/8/2018
Venetoclax (Venclexta)	Newly diagnosed AML	11/21/2018	10/16/2020

Abbreviations: AA, accelerated approval; AIDS, acquired immunodeficiency syndrome; ALK, anaplastic lymphoma receptor tyrosine kinase; ALL, acute lymphocytic leukemia; AML, Acute Myeloid Leukemia; AP, accelerated phase; ASCT, autologous stem cell transplant; BCC, basal cell carcinoma; BC, breast cancer; BP, blast phase; BRAF, B-Raf proto-oncogene, serine/threonine kinase; HL, Hodgkin Lymphoma; CLL, chronic lymphocytic leukemia; CML, chronic myelogenous leukemia; CRC, colorectal cancer; CP, chronic phase; CTCL, cutaneous T-cell lymphoma; EC, endometrial cancer; EGFR, epidermal growth factor receptor; DLBCL, diffuse large B-cell lymphoma; dMMR, mismatch repair deficient; EGFR, epidermal growth factor receptor; FRα, folate receptor-alpha; GIST, gastrointestinal stromal tumor; FGFR2, fibroblast growth factor receptor 2; FGFR3, fibroblast growth factor receptor 3; HCC, hepatocellular carcinoma; HER2, human epidermal growth factor receptor 2; HL, Hodgkin lymphoma; HNSCC, head and neck squamous cell carcinoma; HR, hormone receptor; MCC, Merkel cell carcinoma; MET, mesenchymal-epithelial transition; MM, multiple myeloma; MRD, minimal residual disease; MSI-H, microsatellite instability-high; MTC, medullary thyroid cancer; NHL, non-Hodgkin lymphoma; NSCLC, non–small cell lung cancer; PD-L1, programmed death-ligand 1; Ph, Philadelphia chromosome; RA, regular approval; RCC, renal cell carcinoma; SEGA, subependymal giant cell astrocytoma; TNBC, Triple-Negative Breast Cancer; TSC, tuberous sclerosis complex; UC, urothelial carcinoma.

© 2025 Tibau A et al. *JAMA Network Open*.

Supplementary Table 2. Accelerated Approvals for Malignant Hematology and Oncology Products That Were Withdrawn

**Product**	**Original Accelerated Approval Indication**	**Date of AA**	**Date of Withdrawn**
Atezolizumab (Tecentriq)	Refractory advanced UC	5/18/2016	4/13/2021
Atezolizumab (Tecentriq)	Advanced UC ineligible for cisplatin-based chemotherapy with PD-L1+ or ineligible for any platinum-based chemotherapy, regardless of PD-L1	4/17/2017	12/2/2022
Atezolizumab (Tecentriq)	Newly diagnosed locally advanced or metastatic PD-L1+ TNBC	3/8/2019	10/6/2021
Belantamab mafodotin-blmf (Blenrep)	Refractory MM	8/5/2020	2/6/2023
Bevacizumab (Avastin)	Newly diagnosed metastatic HER2- BC	2/22/2008	11/18/2011
Celecoxib (Celebrex)	To decrease adenomatous colorectal polyps in familial adenomatous polyposis patients.	12/23/1999	6/8/2012
Copanlisib (Aliqopa)	Refractory FL	9/14/2017	3/18/2024
Durvalumab (Imfinzi)	Refractory locally advanced or metastatic UC	5/1/2017	2/19/2021
Duvelisib (Copiktra)	Refractory FL	9/24/2018	12/17/2021
Fludarabine phosphate (Oforta)	Refractory B-cell CLL	12/18/2008	12/31/2011
Gefitinib (Iressa)	Refractory locally advanced or metastatic NSCLC	5/5/2003	4/25/2012
Gemtuzumab ozogamicin (Mylotarg)	Refractory CD33+ AML aged 60 years or older	5/17/2000	11/28/2011
Ibrutinib (Imbruvica)	Refractory MCL	11/13/2013	5/18/2023
Ibrutinib (Imbruvica)	Refractory MZL	1/18/2017	5/18/2023
Idelalisib (Zydelig)	Refractory follicular B-cell NHL and SLL	7/23/2014	2/18/2022
Infigratinib (Truseltiq)	Refractory advanced cholangiocarcinoma with FGFR2 gene fusions	5/28/2021	5/16/2024
Melphalan flufenamide (Pepaxto)	Refractory MM	2/26/2021	2/23/2024
Mobocertinib (Exkivity)	Refractory advanced NSCLC with EGFR exon 20 insertions	9/15/2021	7/15/2024
Nivolumab (Opdivo)	Refractory HCC	9/22/2017	7/23/2021
Nivolumab (Opdivo)	Refractory metastatic SCLC	8/16/2018	12/29/2020
Olaratumab (Lartruvo)	Advanced soft tissue sarcoma	10/19/2016	2/25/2020
Panobinostat (Farydak)	Refractory MM	2/23/2015	3/24/2022
Pembrolizumab (Keytruda)	Refractory locally advanced or metastatic PD-L1+ gastric or GEJ adenocarcinoma	9/22/2017	2/4/2022
Pembrolizumab (Keytruda)	Refractory metastatic SCLC	6/17/2019	3/30/2021
Pralsetinib (Gavreto)	Advanced or metastatic RET-mutant MTC	12/1/2020	7/20/2023
Romidepsin (Istodax)	Refractory peripheral T-cell lymphoma	6/16/2011	7/30/2021
Tositumomab and [^{131}I] tositumomab (Bexxar)	Refractory low-grade follicular or transformed CD20+ NHL	12/22/2004	10/23/2013
Umbralisib (Ukoniq)	Refractory MZL	2/5/2021	5/31/2022

© 2025 Tibau A et al. *JAMA Network Open*.

Umbralisib (Ukoniq)	Refractory FL	2/5/2021	5/31/2022
Vincristine sulfate liposomal (Marqibo)	Refractory Ph- ALL	8/9/2012	5/2/2022
Abbreviations: AA, accelerated approval; ALL, acute lymphocytic leukemia; AML, Acute Myeloid Leukemia; BC, breast cancer; CLL, chronic lymphocytic leukemia; FGFR2, fibroblast growth factor receptor 2; EGFR, epidermal growth factor receptor; FL, follicular lymphoma; HCC, hepatocellular carcinoma; HER2, human epidermal growth factor receptor 2; MCL, mantle cell lymphoma; MM, multiple myeloma; MZL, marginal zone lymphoma; MTC, medullary thyroid cancer; NHL, non-Hodgkin lymphoma; NSCLC, non–small cell lung cancer; PD-L1, programmed death-ligand 1; Ph, Philadelphia chromosome; RA, regular approval; SCLC, small cell lung cancer; SLL, small lymphocytic lymphoma; TNBC, Triple-Negative Breast Cancer; UC, urothelial carcinoma.			

© 2025 Tibau A et al. *JAMA Network Open*.

eTable 3. Factors at Accelerated Approval Associated with Time to Full Approval and Withdrawal for Anticancer Drugs, 1992-2022

		Oncology Indications Converted to Regular Approval or Withdrawn		
		FDA Approval and Withdrawal	Differences in approval times, years	P-value[a]
**Cancer drug indications and pivotal trials**		**n (%)**	**Median (IQR)**	
		132 (100)		
Priority Review [b]		132 (100)		**0.002**
	Yes	107 (81)	3.03 (2.11-4.54)	
	No	25 (19)	6.33 (3.12-8.23)	
Breakthrough Therapy designation[c]		82 (100)		0.67
	Yes	45 (55)	2.60 (1.82-4.09)	
	No	37 (45)	2.97 (1.82-4.09)	
Orphan Drug Act designation[d]		132 (100)		0.11
	Yes	87 (xx)	3.03 (1.84-4.66)	
	No	45 (34)	3.40 (2.33-6.33)	
Tumor Type		132 (100)		**0.012**
	Solid Cancer	81 (61)	3.03 (2.01-4.46)	
	Hematologic Cancer	51 (39)	3.96 (2.50-7.54)	
Type of indication		132 (100)		0.08
	Initial	79 (60)	2.97 (2.15-4.77)	
	Supplemental	53 (40)	4.22 (2.35-5.54)	
Companion diagnostic		132 (100)		0.051
	Yes	43 (32)	2.84 (2.24-4.10)	
	No	90 (68)	3.74 (2.17-6.41)	
Genome-targeted drugs		132 (100)		0.14
	Yes	41 (31)	2.93 (2.25-4.16)	
	No	91 (69)	3.64 (2.15-6.33)	
Immunotherapy		132 (100)		0.31
	Yes	31 (23)	3.34 (1.84-4.54)	
	No	101 (77)	3.23 (2.20-6.35)	
Number of trials supporting approval		132 (100)		0.66
	1	103 (78)	3.34 (2.21-4.90)	
	>1	29 (22)	3.07 (1.90-7.97)	
Study design		132 (100)		0.73
	SAT	43 (33)	3.14 (2.21-5.24)	
	RCT	89 (67)	3.43 (2.15-5.50)	
Blinding		43 (100)		0.64
	Open-label	33 (77)	3.40 (1.86-5.77)	
	Double-blind	10 (23)	3.62 (2.92-5.90)	
Time to event endpoint leading		132 (100)		0.53
	Yes	20 (15)	3.23 (1.60-5.43)	
	No	112 (85)	3.28 (2.22-5.24)	
Confirmatory trial ongoing at time of accelerated approval		132(100)		**<0.001**
	Yes	100 (76)	2.98 (1.88-4.36)	

© 2025 Tibau A et al. *JAMA Network Open*.

	No	32 (24)	5.98 (3.25-8.54)	
Boxed warnings		132 (100)		**<0.001**
	Yes	37 (28)	4.61 (2.72-8.23)	
	No	95 (72)	3.03 (1.87-4.51)	
Low clinical benefit ESMO-MCBS		130 (100)		**0.009**
	Yes	86 (66)	3.81 (2.50-6.37)	
	No	44 (34)	2.58 (1.90-3.99)	
	**Median (IQR)**	**FDA Approval**	**Spearman's Rho**	**P-value[c]**
Number of patients	132 (86.25-217.25)	132	-0.15	0.09
Response Rate	33.50 (22.5-54)	102	-0.09	0.29
Number of Warnings and Precautions	7 (4-9)	132	0.16	0.07

[a] The Mann-Whitney U test assessed whether application and trial characteristics in the accelerated approval pathway were associated with time from accelerated approval to full approval or withdrawal. Bold indicates statistically significant P value.

[b] Breakthrough therapy designation came into effect in July 2012

[c] Spearman's Rho evaluated correlations between continuous variables and time to demonstrate or refute clinical benefit.

[d] ESMO-MCBS was applicable to 130 of the 132 trials (98%). Among these, 13 trials (10%) demonstrated a high clinical benefit; 31 trials (24%) intermediate, and 86 trials (66%) low clinical benefit.

Abbreviation: ESMO-MCBS, European Society for Medical Oncology Magnitude of Clinical Benefit; FDA, the Food and Drug Administration of the United States; IQR, interquartile range.

© 2025 Tibau A et al. *JAMA Network Open*.

eTable 4. Clinical Benefit of Confirmatory Trials Associated with Time to Full Approval and Withdrawal for Anticancer Drugs Granted Accelerated Approval, 1992–2022.				
		**FDA Approval and Withdrawal**	**Differences in approval times, years**	**P-value [a]**
**Confirmatory Trials**		**n (%)**	**Median (IQR)**	
Overall survival benefit[b]		132 (100)		**<0.001**
	Yes	34 (26)	2.15 (1.40-3.38)	
	No	98 (74)	3.81 (2.47-6.44)	
Quality of life benefit		38 (100)		**0.026**
	Yes	15 (39)	2.34 (1.86-3.59)	
	No	23 (61)	4.22 (2.66-5.59)	
ESMO-MCBS clinical benefit		128 (100)		**<0.001**
	Yes	46 (36)	2.34 (1.52-3.39)	
	No	82 (64)	3.85 (2.62-6.53)	

[a] The Mann-Whitney U test evaluated whether overall survival, quality of life, and ESMO-MCBS clinical benefit were associated with time from accelerated to full approval or withdrawal. Statistically significant P values are in bold.

[b] Overall survival benefit as documented in FDA-approved labeling.

[c] Quality of life data were extracted from articles reporting confirmatory trial results. At the time of regular approval conversion, only 38 pivotal trials included quality-of-life data.

[d] ESMO-MCBS was applicable to 128 of the 132 trials (96%). Withdrawn indications were categorized as having low benefit. Among these, 46 trials (36%) demonstrated a high clinical benefit; 29 trials (22%) intermediate, and 53 trials (41%) low clinical benefit.

Abbreviation: ESMO-MCBS, European Society for Medical Oncology Magnitude of Clinical Benefit; FDA, the Food and Drug Administration of the United States; IQR, interquartile range.

© 2025 Tibau A et al. *JAMA Network Open*.
