# Supplementary material for: Factors in Time to Full Approval or Withdrawal for Anticancer Medicines Granted Accelerated Approval by the FDA
Source: JAMA Netw Open. 2025 Mar 26;8(3):e252026. doi: 10.1001/jamanetworkopen.2025.2026 (PMC11947834; doi:10.1001/jamanetworkopen.2025.2026)
Supplement: Supplement 2. — Data Sharing Statement [file jamanetwopen-e252026-s002.pdf]

Data Sharing Statement

Tibau. Factors in Time to Full Approval or Withdrawal for Anticancer Medicines Granted Accelerated Approval by the FDA. *JAMA Netw Open*. Published March 26, 2025. doi:10.1001/jamanetworkopen.2025.2026

Data
Data available: Yes
Data types: Data (not involving human participants)
How to access data: All the data is available online, but it can also be provided via email at atibaumartorell@bwh.harvard.edu.
When available: With publication

Supporting Documents
Document types: Statistical/analytic code
How to access documents: This data can be provided via email at atibaumartorell@bwh.harvard.edu.
When available: With publication

Additional Information
Who can access the data: To anyone requesting the data
Types of analyses: For a specified purpose
Mechanisms of data availability: After approval of a proposal
Any additional restrictions: N/A
